# Anatomic Study of Anterior Transdiscal Axial Screw Fixation for Subaxial Cervical Spine Injuries

**DOI:** 10.1097/MD.0000000000003723

**Published:** 2016-08-07

**Authors:** Wei Ji, Minghui Zheng, Dongbin Qu, Lin Zou, Yongquan Chen, Jianting Chen, Qingan Zhu

**Affiliations:** From the Department of Spinal Surgery, Nanfang Hospital, Southern Medical University, Guangzhou, China.

## Abstract

Anterior transdiscal axial screw (ATAS) fixation is an alternative or supplement to the plate and screw constructs for the upper cervical spine injury. However, no existing literatures clarified the anatomic feasibility of this technique for subaxial cervical spine. Therefore, the objective of this study was to evaluate the anatomical feasibility and to establish guidelines for the use of the ATAS fixation for the subaxial cervical spine injury.

Fifty normal cervical spines had radiographs to determine the proposed screw trajectory (the screw length and insertion angle) and the interbody graft-related parameters (the disc height and depth, and the distance between anterior vertebral margin and the screw) for all levels of the subaxial cervical spine. Following screw insertion in 8 preserved human cadaver specimens, surgical simulation and dissection verified the feasibility and safety of the ATAS fixation.

Radiographic measurements showed the mean axial screw length and cephalic incline angle of all levels were 41.2 mm and 25.2°, respectively. The suitable depth of the interbody graft was >11.7 mm (the distance between anterior vertebral margin and the screw), but <17.1 mm (disc depth). Except the axial screw length, increase in all the measurements was seen with level up to C5–C6 segment. Simulated procedure in the preserved specimens demonstrated that ATAS fixation could be successfully performed at C2–C3, C3–C4, C4–C5, and C5–C6 levels, but impossible at C6–C7 due to the obstacle of the sternum. All screws were placed accurately. None of the screws penetrated into the spinal canal and caused fractures determined by dissecting the specimens.

The anterior transdiscal axial screw fixation, as an alternative or supplementary instrumentation for subaxial cervical spine injuries, is feasible and safe with meticulous surgical planning.

## INTRODUCTION

Cervical flexion distraction injuries account for about 10% of all injuries to the subaxial cervical spine and mainly caused by traffic accidents.^1,2^ The injuries involve not only the intervertebral disc and anterior ligament, but also posterior complex ligament, which may further result in subluxation or dislocation of the facet joint.^3–7^ The instability associated with cervical flexion distraction injury is emphasized due to those circumferential injuries affecting both anterior and posterior structures.

Although some surgeons proposed conservative treatment for the patients suffered mild flexion distraction injury,^8–10^ more authors recommend aggressive surgical treatment would be one choice.^11–13^ In most cases, anterior cervical decompression and fusion (ACDF) plus plate instrumentation was carried out according to the injury mechanism and pathology. But Johnson et al^14^ reported that 13% of cases received anterior cervical procedure alone would result in cervical deformity healing. It was considered that only anterior cervical plate fixation could not supply sufficient biomechanical stability in those cases with severe facet fracture and posterior complex injury.^15^ More authors recommended that the combined anterior and posterior approach should be applied, that means, on the basis of ACDF and anterior plate, the posterior screw-rods instrumentation should also be added.^16–18^ However, the complications associated with those combined procedures were also noted with high risks of surgical trauma.

In 2006, Defino et al^19^ put forward a procedure for anterior C2/3 screw fixation and performed a biomechanical study in swine cervical spine. The results showed that anterior 2 axial screws for C2/3 fixation could achieve similar biomechanical stability compared to anterior cervical plate fixation. Therefore, the author suggested that anterior transdiscal axial screw fixation (ATAS) of C2–3 could be considered as an alternative procedure if some difficulty of exposing or instrumenting occurred when performing the anterior plate fixation.

Given these potential uses for anterior transdiscal axial screw fixation, we believe that this technique should be included in the armamentarium of subaxial cervical spine fixation methods. And yet, to the best of our knowledge, there are no literatures that have document the detailed and relative anatomical data for ATAS. In this study, the authors proposed the ATAS fixation technique as a supplementary instrumentation for the subaxial cervical spine injury. The anatomic feasibility was assessed through a radiographic study of patients and an evaluation of human cadaveric spine specimens.

## MATERIALS AND METHODS

### Radiographic Measurements

This was an institutional review board-approved, retrospective analysis of patients of East Asian ancestry who presented to the emergency department at Nanfang hospital between January 1, 2015, and July 30, 2015, requiring standard lateral x-ray films of the cervical spine. To be included, the films have to be with normal cervical curve and alignment, to be negative for the presence of endplate sclerosis, osteophytes, and narrowing of disc spaces or segmental instability. Fifty patients (32 males/18 females, range 22–48 years) with mean age of 28 ± 4 years were available for analysis. The lateral films of the cervical spine were obtained from a 500-mA x-ray machine (Siemens, Erlangen, Germany) and the lines and angles were drawn and measured using PACS (INFINIFF, Seoul, South Korea).

**FIGURE 1:**
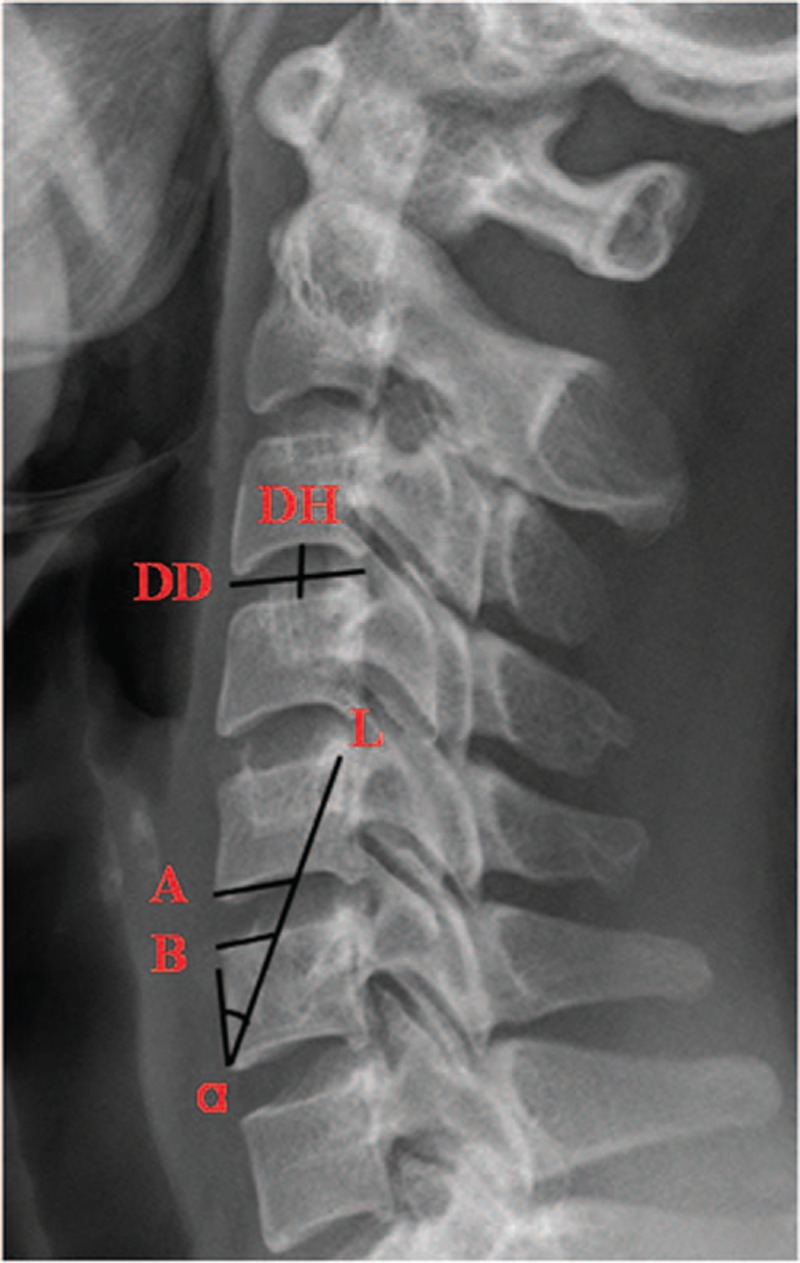
Measurement of the parameters related with ATAS fixation. α = the angle between the simulated trajectory of the axial screw and the anterior edge of the vertebral body, A = the depth of the superior border of the graft, ATAS = anterior transdiscal axial screw, B = the depth of the inferior border of the graft, DD = disc depth, DH = disc height, L = axial screw length.

On the lateral x-ray film, the following parameters related with ATAS were measured (Figure 1): (L) axial screw length, made from anteroinferior point of inferior vertebral body to the posterosuperior point of superior vertebral body (the diagonal line of the adjoining cervical vertebral bodies); (α) cephalic incline angle of axial screw, made between the simulated trajectory of the screw and the front edge of the vertebral body; (DH) disc height, the height of the intervertebral space; (DD) disc depth, the distance between the anterior edge and posterior edge of the cervical disc; (A) graft depth A, the length between the intersection of the trajectory of the axial screw with the inferior endplate of superior vertebral body and its anterior inferior border, representing the depth of the superior border of the graft; (B) graft depth B, the length between the intersection of the trajectory of the axial screw with the superior endplate of inferior vertebral body and its anterior inferior border, representing the depth of the inferior border of the graft. The parameters measurement of the ATAS were performed at C2–C3, C3–C4, C4–C5, and C5–C6 levels, but the C6–C7 level in all patients, due to the obstruction of the sternum.

### Cadaveric Simulation

**FIGURE 2:**
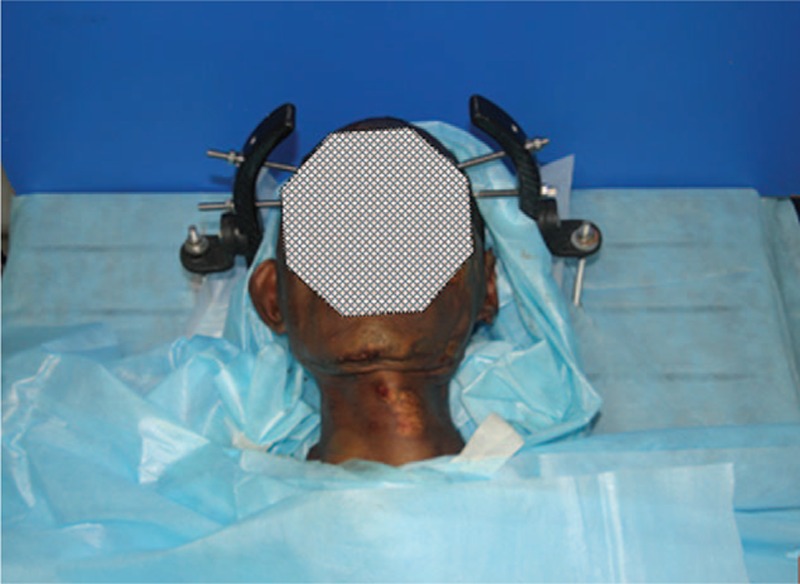
Photograph of anatomical preparation of a cadaveric head on a head holder.

**FIGURE 3:**
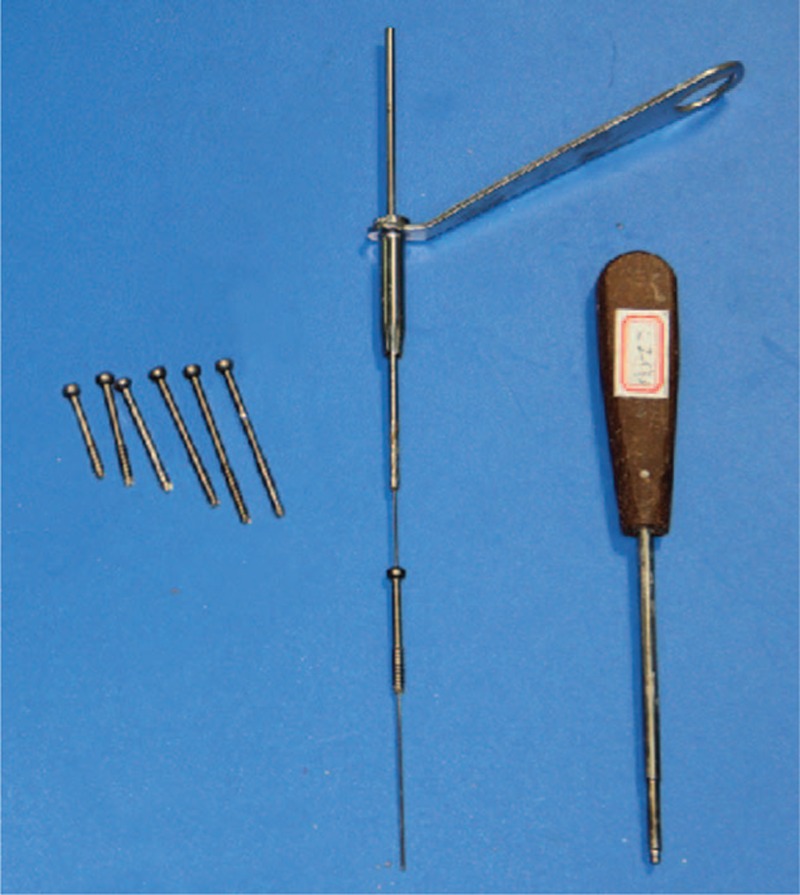
Photograph showing the system of tools to facilitate insertion of screw.

Eight preserved human cadaver specimens (5 males/3 females) of East Asian ancestry with unknown age were obtained from the Department of Anatomy. The specimen was confirmed by x-ray to have a normal cervical spine. The cadaver heads were secured with pins in a head-holder (Figure 2), avoiding flexion-extension and rotation. The tools necessary to facilitate insertion of a screw include the following equipment seen in Figure 3.

The surgical procedure of ATAS fixation was carried out as follows. The midline of vertebral body was confirmed and used as guidance for axial screw insertion. Under the lateral C-arm x-ray fluoroscope, guiding wire was inserted from the anteroinferior portion of the inferior cervical vertebral body, pointing to the posterosuperior portion of the superior cervical vertebral body. After the guiding wire positioned successfully, the trajectory length of the wire was measured on the surplus distance by a second identical K-wire placed parallel with the initial guiding wire. The screw length was determined based on the length of the wire trajectory. A cannulated drill bit was used to make a recess for the screw insertion. Then, a partially threaded self-tapping cannulated screw (diameter in 3.5 or 4.0 mm) was inserted over the guiding wire. Once the axial screw was successfully positioned, the wire was then removed. The 8 specimens were dissected to observe the incidence of violation to the spinal canal and vertebral fracture after the ATAS fixation.

## STATISTICAL ANALYSIS

All collected data were further processed with SPSS software (SPSS Inc, Chicago, IL), and expressed in mean ± standard deviation. A 1-way analysis of variance was used to compare all parameters among levels. The significance level was set at 95%.

## RESULTS

### Radiographic Measurements

Given that no significant difference was observed (*P* < 0.05) in the measurements for sexes, the results presented here correspond to averages obtained without considering the sexes (Tables 1 and 2). Except the axial screw length, increase in all the measurements was seen with level up to the C5–C6 segment.

**TABLE 1:**
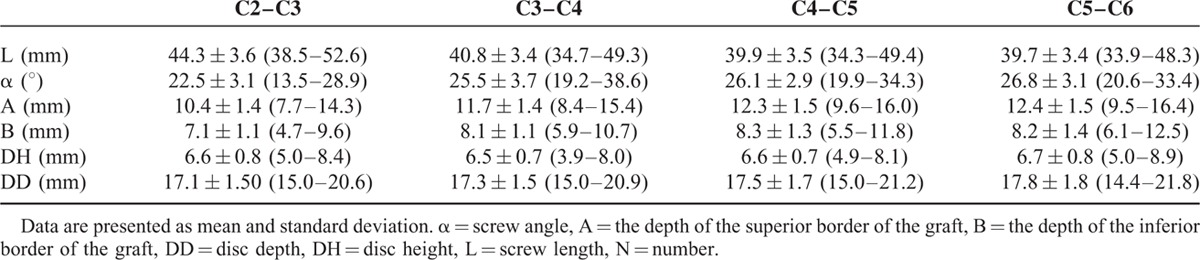
Measurement Parameters Related With Anterior Transdiscal Axial Screw Fixation (Patients N = 50)

**TABLE 2:**
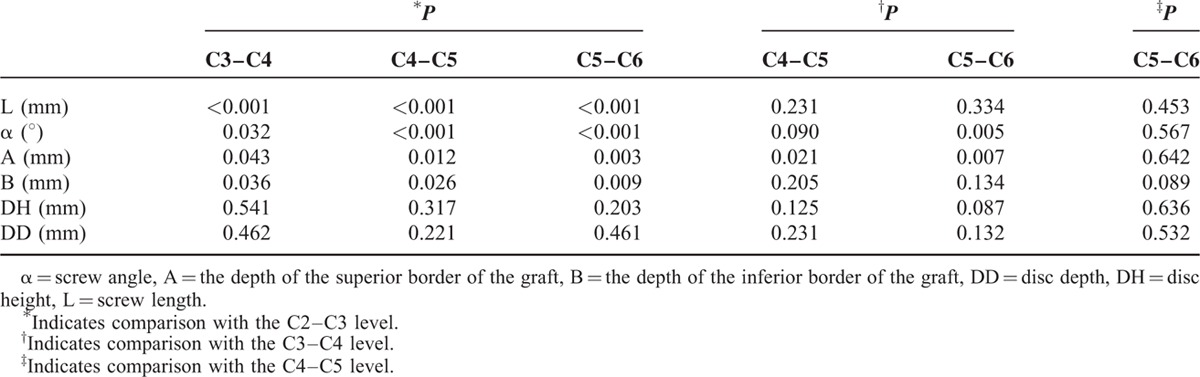
*P* Values of Post Hoc Analysis Between Levels for all Measurements

The mean screw length and angle for all patients were 41.2 mm and 25.2°, respectively. The C2–C3 segment has the longest axial screw, but with a smallest screw inclination angle. The mean graft depth A and B for all patients were 11.7 mm and 7.9 mm, respectively. Increase in the both measurements was seen with level up to the C5–C6 segment. There was a significant difference between levels in graft depth A, except for C4–C5 and C5–C6 levels. In contrast, in graft depth B, no significant difference was observed between levels except for C2–C3 and the other levels. The mean disc height and disc depth for all patients were 6.6 mm and 17.4 mm, respectively. The above both parameters also appear to be a growing trend with level up. There were no significance differences between the levels for the disc height or disc depth (Table 2).

### Simulation Procedure

**FIGURE 4:**
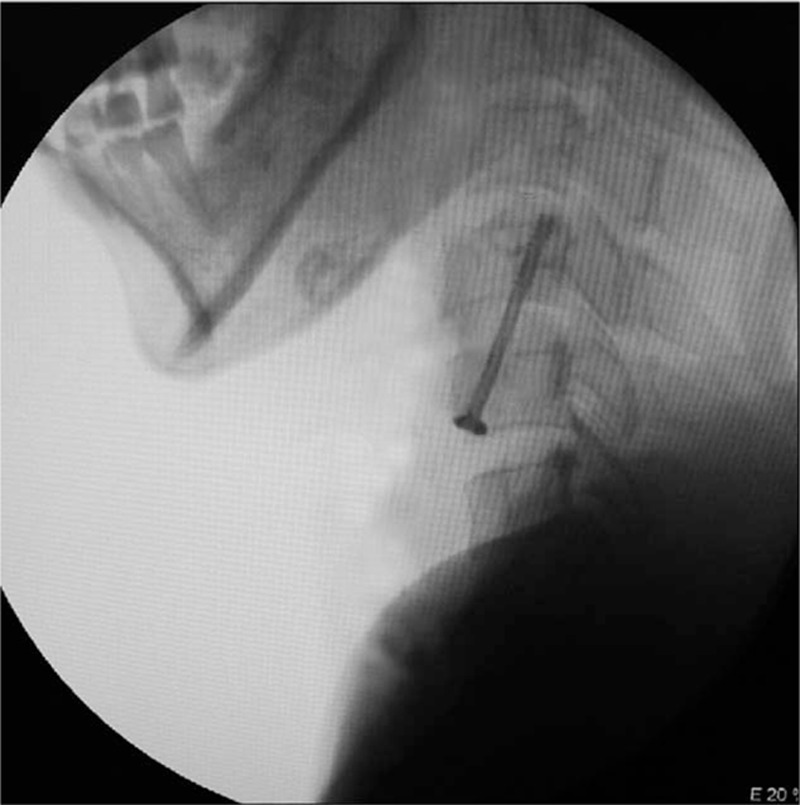
X-ray image of ATAS fixation. A partially threaded self-tapping cannulated screw with an upper oblique angle, passing through the lower vertebral body and the intervertebral space into the upper adjacent vertebral body. ATAS = anterior transdiscal axial screw.

ATAS fixation was performed successfully at C2–C3, C3–C4, C4–C5, and C5–C6 segments in all specimens (Figure 4). Due to the obstruction of the sternum, however, it could not be conducted in the C6–C7 segment. On gross examination, all 32 screws met the criteria for viable positioning. Based on dissection of the specimens, none of screws penetrated into the spinal canal and caused vertebral fracture.

## DISCUSSION

Anterior axial fixation was first introduced in the lumbosacral zone with a fibular strut or direct screw fixation, which was introduced in the midline from the anterior superior border of L5, obliquely down through the vertebral body of L5 and the disk space L5/S1, into the vertebral body of S1.^20,21^ The procedure was recommended for the treatment of lumbosacral spondylolisthesis. Recently, some reports presented a posterior axial fixation technique by using the transpedicle-disc-vertebral body screw for the lumbosacral junction.^22–24^ Furthermore, this procedure also can achieve a delta fixation combined with other pedicle screw and rod application. Aebi et al^21^ noted that the procedure was simple and easy to perform, and the overall satisfaction of the patients was >80%. It was anticipated that the instant stability supported by the axial screw would be superior to the vertebral cancellous screw fixation such as the anterior plate-screw system. But all these fixation methods are not the mainstream spinal instrumentation techniques clinically.

In 2004, Cragg et al^25^ first introduced a new procedure of percutaneous axial lumbosacral interbody fusion (AxiaLIF). With a presacral working tunnel through a small incision in paracoccyx, 1 specially designed screw was introduced from anterior S1 to L5, or L4 for the lumbosacral fixation. Erkan et al^26^ conducted a biomechanical study of 2-level fusion of L4-S1 AxiaLIF in 6 lumbosacral cadaveric specimens. Three states including the AxiaLIF alone, AxiaLIF with posterior facet screws, and AxiaLIF with pedicle screws and rods fixation were evaluated, respectively. The result indicated that at the L4–L5 level, there was no statistical significance in rotation or flexion-extension movement among the 3 different AxiaLIF procedures, and the AxiaLIF with pedicle screws and rods fixation shows a significantly higher stability than the other 2 fixation methods in lateral bending movement; at the L5–S1 level, there was no significance difference in rotation and lateral bending movement among the 3 fixation methods, and the AxiaLIF alone showed a larger range of motion in flexion-extension, the AxiaLIF with posterior constructs fixation had an increased stiffness in this level. Ledet et al^27^ reported that the axial screw fixation had more segmental stability than the traditional implants such as intervertebral cage, thus promoting bony fusion. Initial clinical experience on AxiaLIF showed this procedure had good effects on lumbosacral fusion in early stage.^28^

For upper cervical spine, axial screw fixation was commonly used as posterior or anterior atlantoaxial transarticular fixation. Magerl and Seemann ^29^ first introduced the posterior transarticular screw fixation for atlantoaxial instability. More authors supported this procedure can apply sufficient biomechanical stability, and >90% of fusion rate can be achieved by this procedure. Anterior atlantoaxial transarticular fixation was introduced by Barbour,^30^ and the clinical results confirming the superior efficacy of this technique.

To our knowledge, the ATAS fixation is not yet reported as the normal surgical procedure for the subaxial cervical spine injury in the literature. Argenson et al^31^ showed us some figures of fixation by 1 anterior screw and graft between C3 and C2 in the case with severe sprain injury. In 2006, Defino et al^19^ put forward a procedure for anterior C2–C3 screw fixation and performed a biomechanical study in swine cervical spine. The results showed that anterior 2 axial screws for C2/3 fixation could achieve similar biomechanical stability compared to anterior cervical plate fixation. Therefore, the author suggested that the axial screw fixation of C2–C3 could be considered as an alternative procedure if some difficulty of exposing or instrumenting occurred when performing the anterior plate fixation.

The present study was the first anatomical analysis of the ATAS fixation. The important parameters related to this fixation were collected from this study. For adult, about 41-mm length cancellous screw was adapted for most cases, and the preferable inclination angle was nearly 25°. Simulation surgery showed that the axial screw fixation can be carried out for C2–C3, C3–C4, C4–C5, and C5–C6 levels. For the C6–C7 level, cephalically axial screw was not possible due to the obstruction of the sternum. Therefore, the ATAS fixation was feasible and safe to perform.

Theoretically, biomechanical advantages of ATAS fixation were noted as following aspects. First, the length of the anterior cervical axial screw (41 mm) was remarkable longer than that of the commercially designed cervical plate screw (12–18 mm), which should favor resistance to screw pull-out. Second, ATAS as a fixation procedure of vertebral body-disc-body could penetrate at least 2 endplate structures and achieved more biomechanical benefits compared to the simple cancellous screw. Luk et al^32^ evaluated biomechanically the insertion torque and maximal pull-out strength between conventional sacral pedicle screw and the bicortical sacral pedicle screw through the S1 endplate and confirmed the superiority of the screw fixation with the trajectory through the S1 endplate. Third, anterior cervical plate fixation only had the tension-band effect anteriorly, and the flexion stability was insufficient under complete destruction of the posterior elements. Supplementary anterior axial screw could add the beneficial synergistic effect with anterior plate instrumentation for the treatment of the cervical instability. With ATAS, the adjoining vertebral bodies and in-between graft were hold together as a whole, which can be seen as a biomechanical fusion.

ATAS fixation is recommended for the cervical flexion distraction or rotational injury, which results in transdisc fracture or dislocation. Although this fixation technique has the same dissection plane as the anterior plate and screw fixation system, the exposure area for ATAS placement is smaller compared to the standard technique, involving a lower risk of injury to adjacent structures. When the anterior plate and screw fixation system could not provide sufficient stability or the procedure is impossible to perform in the cervical spine, the availability of the ATAS fixation technique may add stable fixation to further attempts at obtaining an anterior spinal fusion. The possible disadvantage of this technique would be the degeneration of the adjacent disc that is sometimes seen in anterior odontoid fixation. As a supplementary anterior instrumentation procedure, the ATAS fixation technique is not suitable for the cases with serious vertebral body fracture.

Some limitations of this study must be acknowledged. For the radiographic study, the sternum cannot be observed on the standard lateral x-ray films of the cervical spine even on the computed tomographic images, and the parameters related to the ATAS fixation were just measured in C2–C3, C3–C4, C4–C5, and C5–C6 segments. Next, for the cadaveric simulation and evaluation, it cannot represent the situation in vivo absolutely. Extending backward of the neck in vivo, the ATAS fixation would be successfully performed in the C6–C7 level in some cases. Finally, this study mainly focused on bony structures and gave less consideration to soft tissue such as a thick fat layer cover the sternum.

## CONCLUSION

The anterior transdiscal axial screw fixation, as an alternative or supplementary instrumentation for the subaxial cervical spine injury, is feasible and safe with meticulous surgical planning. However, further biomechanical studies are required to compare its reliability to other more established techniques.

## References

[1] Vaccaro AR Cook CM McCullen G Cervical trauma: rationale for selecting the appropriate fusion technique. *Orthop Clin North Am* 1998; 29:745–754. 9756969 10.1016/s0030-5898(05)70045-6

[2] Radcliff K Thomasson BG Flexion-distraction injuries of the subaxial cervical spine. *Sem Spine Surg* 2013; 25:45–56.

[3] Berrington NR van Staden JF Willers JG Cervical intervertebral disc prolapse associated with traumatic facet dislocations. *Surg Neurol* 1993; 40:395–399. 8211656 10.1016/0090-3019(93)90219-q

[4] Doran SE Papadopoulos SM Ducker TB Magnetic resonance imaging documentation of coexistent traumatic locked facets of the cervical spine and disc herniation. *J Neurosurg* 1993; 79:341–345. 8360729 10.3171/jns.1993.79.3.0341

[5] Eismont FJ Arena MJ Green BA Extrusion of an intervertebral disc associated with traumatic subluxation or dislocation of cervical facets. Case report. *J Bone Joint Surg Am* 1991; 73:1555–1560. 1748703

[6] Hadley MN Fitzpatrick BC Sonntag VK Facet fracture-dislocation injuries of the cervical spine. *Neurosurgery* 1992; 30:661–666. 1584374

[7] Lee AS MacLean JC Newton DA Rapid traction for reduction of cervical spine dislocations. *J Bone Joint Surg Br* 1994; 76:352–356. 8175833

[8] Olerud C Jonsson H Jr Compression of the cervical spine cord after reduction of fracture dislocations. Report of 2 cases. *Acta Orthopaedica Scandinavica* 1991; 62:599–601. 1767657 10.3109/17453679108994506

[9] Robertson PA Ryan MD Neurological deterioration after reduction of cervical subluxation. Mechanical compression by disc tissue. *J Bone Joint Surg Br* 1992; 74:224–227. 1544957 10.1302/0301-620X.74B2.1544957

[10] Cotler JM Herbison GJ Nasuti JF Closed reduction of traumatic cervical spine dislocation using traction weights up to 140 pounds. *Spine* 1993; 18:386–390. 8475443 10.1097/00007632-199303000-00015

[11] Aebi M Surgical treatment of upper, middle and lower cervical injuries and non-unions by anterior procedures. *Eur Spine J* 2010; 19:S33–S39. 19826842 10.1007/s00586-009-1120-8 PMC2899722

[12] Aebi M Zuber K Marchesi D Treatment of cervical spine injuries with anterior plating. Indications, techniques, and results. *Spine* 1991; 16:S38–S45. 2028339 10.1097/00007632-199103001-00008

[13] Razack N Green BA Levi AD The management of traumatic cervical bilateral facet fracture-dislocations with unicortical anterior plates. *J Spinal Disord* 2000; 13:374–381. 11052345 10.1097/00002517-200010000-00002

[14] Johnson MG Fisher CG Boyd M The radiographic failure of single segment anterior cervical plate fixation in traumatic cervical flexion distraction injuries. *Spine* 2004; 29:2815–2820. 15599284 10.1097/01.brs.0000151088.80797.bd

[15] Paxinos O Ghanayem AJ Zindrick MR Anterior cervical discectomy and fusion with a locked plate and wedged graft effectively stabilizes flexion-distraction stage-3 injury in the lower cervical spine: a biomechanical study. *Spine* 2009; 34:E9–E15. 19127153 10.1097/BRS.0b013e318188386a

[16] Song KJ Lee KB Anterior versus combined anterior and posterior fixation/fusion in the treatment of distraction-flexion injury in the lower cervical spine. *J Clin Neurosci* 2008; 15:36–42. 18061456 10.1016/j.jocn.2007.05.010

[17] Do Koh Y Lim TH Won You J A biomechanical comparison of modern anterior and posterior plate fixation of the cervical spine. *Spine* 2001; 26:15–21. 11148640 10.1097/00007632-200101010-00005

[18] Tofuku K Koga H Yone K Distractive flexion injuries of the subaxial cervical spine treated with a posterior procedure using cervical pedicle screws or a combined anterior and posterior procedure. *J Clin Neurosci* 2013; 20:697–701. 23313522 10.1016/j.jocn.2012.03.045

[19] Defino HL Neri OJ Shimano AC Anterior C2-C3 fixation with screws: proposal of a new technique and comparative mechanical assays. *Eur Spine J* 2006; 15:1159–1164. 16841224 10.1007/s00586-005-0011-x PMC3233936

[20] Marchetti PG Binazzi R Briccoli A The surgical treatment of spondylolisthesis. *Chir Organi Mov* 1994; 79:85–91. 8076482

[21] Aebi M Szpalski M Transpedicular-transdiscal-transcorporal (TPDC)-fixation. *Surgery for Low Back Pain*. Berlin, Heidelberg: Springer-Verlag; 2010 147–154.

[22] Bartolozzi P Sandri A Cassini M One-stage posterior decompression-stabilization and trans-sacral interbody fusion after partial reduction for severe L5-S1 spondylolisthesis. *Spine* 2003; 28:1135–1141. 12782981 10.1097/01.BRS.0000067274.38273.5C

[23] Boachi-Adjei O Do T Rawlins BA Partial lumbosacral kyphosis, reduction, decompression and posterior lumbosacral transfixation in high grade isthmic spondylolisthesis: clinical and radiographic results in six patients. *Spine* 2002; 27:E161–E168. 11884921 10.1097/00007632-200203150-00019

[24] Smith JA Deviren V Berven S Clinical outcome of trans-sacral interbody fusion after partial reduction for high grade L5/S1 spondylolisthesis. *Spine* 2001; 26:227–2234. 10.1097/00007632-200110150-00014 11598513

[25] Cragg A Carl A Casteneda F New percutaneous access method for minimally invasive anterior lumbosacral surgery. *J Spinal Disord Tech* 2004; 17:21–28. 14734972 10.1097/00024720-200402000-00006

[26] Erkan S Wu C Mehbod AA Biomechanical evaluation of a new AxiaLIF technique for two-level lumbar fusion. *Eur Spine J* 2009; 18:807–814. 19352729 10.1007/s00586-009-0953-5 PMC2899656

[27] Ledet EH Tymeson MP Salerno S Biomechanical evaluation of a novel lumbosacral axial fixation device. *J Biomech Eng* 2005; 127:929–933. 16438229 10.1115/1.2049334

[28] Aryan HE Newman CB Gold JJ Percutaneous axial lumbar interbody fusion (AxiaLIF) of the L5-S1 segment: initial clinical and radiographic experience. *Minim Invasive Neurosurg* 2008; 51:225–230. 18683115 10.1055/s-2008-1080915

[29] Magerl F Seemann P-S Kehr P Weidner A Stable posterior fusion of the atlas and axis by transarticular screw fixation. *Cervical Spine I*. New York, NY: Springer-Verlag; 1985 322–327.

[30] Barbour JR Screw fixation in fractures of the odontoid process. *S Aust Clinics* 1971; 5:20.

[31] Argenson C de Peretti F Ghabris A Classification of lower cervical spine injuries. *Eur J Orthop Surg Traumatol* 1997; 7:215–219.

[32] Luk KD Chen L Lu WW A stronger bicortical sacral pedicle screw fixation through the s1 endplate: an in vitro cyclic loading and pull-out force evaluation. *Spine* 2005; 30:525–529. 15738784 10.1097/01.brs.0000154649.55589.bf

